# Investigating the impact of electron beam irradiation on electrical, magnetic, and optical properties of XLPE/Co_3_O_4_ nanocomposites

**DOI:** 10.1038/s41598-024-55085-7

**Published:** 2024-02-28

**Authors:** Mohamed Mohamady Ghobashy, A. I. Sharshir, R. A. Zaghlool, F. Mohamed

**Affiliations:** 1 Radiation Research of Polymer Chemistry Department, National Center for Radiation Research and Technology (NCRRT), Egyptian Atomic Energy Authority (EAEA), Cairo, Egypt; 2 Solid State and Electronic Accelerators Department, National Center for Radiation Research and Technology (NCRRT), Egyptian Atomic Energy Authority (EAEA), Cairo, Egypt; 3 Spectroscopy Department, Physics Research Institute, National Research Centre, 33 El Bohouth St., Dokki, 12622 Giza Egypt

**Keywords:** Crosslinked polyethylene, Co_3_O_4_, Nanocomposites, Optical properties, Magnetic properties, Electrical properties, Chemistry, Energy science and technology, Engineering, Materials science, Nanoscience and technology, Optics and photonics, Physics

## Abstract

Nowadays, many researchers aim to fill polymer materials with inorganic nanoparticles to enhance the polymer properties and gain the merits of the polymeric host matrix. Sol–gel synthesized Co_3_O_4_ nanoparticles are subjected to different doses of electron beam (10, 20, and 30 kGy) to study their physiochemical properties and choose the optimized nanoparticles to fill our polymeric matrix. Crosslinked polyethylene (XLPE) has been filled with 5 wt % of un-irradiated cobalt oxide nanoparticles using the melt extruder method. The structural, optical, magnetic, and electrical properties of the XLPE/Co_3_O_4_ nanocomposite before and after exposure to different doses of electron beam radiation have been characterized. The crystallite size of face-centered cubic spinel Co_3_O_4_ nanoparticles has been confirmed by XRD whereas and their unique truncated octahedral shape obviously appears in SEM micrographs. The crystallite size of Co_3_O_4_ nanoparticles has decreased from 47.5 to 31.5 nm upon irradiation at a dose of 30 kGy, and significantly decreased to 18.5 nm upon filling inside XLPE matrix. Related to the oxidation effect of the electron beam, the Co^2+^/Co^3+^ ratio on the surface of Co_3_O_4_ nanoparticles has decreased upon irradiation as verified by XPS technique. This consequently caused the partial elimination of oxygen vacancies, mainly responsible for the weak ferromagnetic behavior of Co_3_O_4_ in its nanoscale. This appears as decreased saturation magnetization as depicted by VSM. The XLPE/Co_3_O_4_ nanocomposite has also shown weak ferromagnetic behavior but the coercive field (H_c_) has increased from 112.57 to 175.72 G upon filling inside XLPE matrix and decreased to 135.18 G after irradiating the nanocomposite at a dose of 30 kGy. The ionic conductivity of XLPE has increased from 0.133 × 10^–7^ to 2.198 × 10^–3^ S/cm upon filling with Co_3_O_4_ nanoparticles while a slight increase is observed upon irradiation.

## Introduction

In recent decades, pursuing advanced materials with tailored properties has driven scientific and technological research^1–7^. Nanocomposites, composed of nanoscale components dispersed within a matrix, have garnered significant attention due to their remarkable combination of properties that can surpass those of their constituents^8–15^. Integrating nanoparticles into polymer matrices has emerged as a promising avenue for achieving enhanced mechanical, electrical, magnetic, and optical characteristics among the diverse range of nanocomposites^16–21^. In particular, incorporating metal oxide nanoparticles into polymers has attracted significant interest in electronics and energy storage applications.

Cross-linked polyethylene (XLPE) is a cost-effective, highly flexible, thermally and chemically stable polymer characterized by good dielectric properties, low conductivity, and easy processing, frequently used as cable insulation material^22–24^. For these merits, filling XLPE with nanoparticles to modify its properties is frequently reported. XLPE/silica nanocomposites were prepared by Sharad et al. using twin screw extruder and injection molding and they proved higher thermal stability upon surface modification by octylsilane^25^. Wang et al. studied the effect of nanoparticle surface modification and filling concentration on space charge characteristics in TiO_2_/XLPE nanocomposites^22^. Madani et al. have also investigated the dielectric behavior of XLPE/BaTiO_3_ nanocomposites in the low-frequency range where the resultant nanocomposite show increased dielectric permittivity and highly decreased loss factor^26^. Mohamed et al. have investigated the electrical treeing behavior in XLPE insulation as it filled with All_2_O_3_ nanoparticles and they observed higher time for treeing growth in XLPE insulation so extended cable life could be achieved^23^. Sharshir et al.^27^ have prepared XLPE and its nanocomposite filled with ZnO nanoparticles which showed improvement of the electric field distribution in medium-voltage cables whereas Wang et al. have verified modified charge transport mechanism of the cable joint as XLPE has doped with MgO nanoparticles^28^. However, no studies are explored on the capability of filling XLPE with magnetic nanoparticles to impart it with magnetic characteristics which would be consequently applied in magnetic systems and spintronics.

Cobalt oxide (Co_3_O_4_) has garnered significant attention among metal oxides due to their intriguing magnetic, catalytic, and electronic properties. Co_3_O_4_ is a reasonably priced^29^ antiferromagnetic^30^ versatile semiconductor^31^ with a unique crystal structure that can influence its electrical and magnetic behavior^32–34^. Co_3_O_4_ can be prepared in nanosized form with controllable shape and dimension with amazing optical and magnetic properties which could be applied in memory devices, electrical switching and microwave devices. In contrast to its bulk form, the nanosized Co_3_O_4_ shows weak ferromagnetism or superparamagnetism^35,36^. In addition to their ferromagnetic behavior, previous studies have highlighted the distinctive attributes of Co_3_O_4_ nanoparticles, including high surface area, and potential for catalytic applications. One intriguing aspect of Co_3_O_4_ is the presence of oxygen vacancies – lattice defects in which oxygen atoms are missing from the crystal structure. Oxygen vacancies can give rise to intriguing phenomena, including alterations in electronic structure, magnetism, and catalytic activity^37–40^. These vacancies introduce localized energy levels within the material's band structure, influencing charge transport and electronic behavior. As a result, oxygen vacancies have garnered significant attention for their potential to modulate material properties and enable new functionalities. So incorporating Co_3_O_4_ nanoparticles into polymer matrices introduces opportunities to engineer and tailor their properties for optical^41^, optoelectronic^42^, electrical, magnetic, and catalysis applications^43,44^.

Many studies on the physical properties of polymer/Co_3_O_4_ nanocomposites have been reported in this context. PVDF/Co_3_O_4_ nanocomposite was prepared by Bhatt et al.^45^ using spincoating method to enhance the polymer’s conductivity and donating magnetic behavior to the matrix. Nandapure et al. have studied the effect of Co_3_O_4_ nanoparticles that prepared using sol–gel method on the magnetic behavior of polyaniline where the PANI/Co_3_O_4_nanocompositewas prepared using in-situ chemical oxidation polymerization of aniline using APS as an oxidant in the presence of Co_3_O_4_ nanoparticles in air at room temperature^46^. Wassel et al. have synthesized Co_3_O_4_ nanoparticles using the thermal decomposition method and investigated the morphological, optical, and magnetic properties of Co_3_O_4_/PANI nanocomposite prepared via in situ oxidative polymerization^47^. PANI/Co_3_O_4_ nanocomposite was also produced by Fan et al.^48^ using in-situ polymerization technique and exhibited great electrochemical performance. Nanocomposites films of PANI-CSA/Co_3_O_4_ NPs were deposited on ITO-glass substrates by Al-Gharram et al. using the electrochemical polymerization method and the structural and optical properties have been investigated^30^. However, preparing XLPE/Co_3_O_4_ nanocomposite and investigating its physical properties have not been explored.

Electron beam irradiation presents a powerful method for inducing controlled structural modifications in materials, offering a means to precisely manipulate properties. Through controlled exposure to electron beams, it is possible to control oxygen vacancies within metal oxide nanoparticles, leading to intriguing changes in material behavior. This introduces a compelling avenue for tuning material properties through the controlled introduction or removal of defects, providing a deeper understanding of the underlying physics and chemistry at the nanoscale^27,49–52^.

In this context, the present study delves into the effects of electron beam irradiation on the structural, optical, and magnetic properties of Co_3_O_4_ nanoparticles, prepared via the sol–gel method, and XLPE/Co_3_O_4_ nanocomposites as well as exploring the conductivity and dielectric properties of XLPE/Co_3_O_4_ nanocomposites. The interplay between these properties and the role of oxygen vacancies, surface cationic states, defects, and crystal lattice distortions are systematically explored.

The outcomes of this research hold significant implications for various technological applications. By tailoring the properties of XLPE/Co_3_O_4_ nanocomposites through controlled electron beam irradiation, the resulting materials can be optimized for specific uses, such as electrical insulation, energy storage, and magnetic devices. Furthermore, the insights gained from this study can contribute to the fundamental understanding of the interactions between nanoparticles and polymers, paving the way for designing advanced nanocomposite materials with tailored functionalities.

## Experimental

### Materials

Cobalt (II) acetate tetrahydrate, with the chemical formula (CH_3_COO)_2_Co·4H_2_O and a molecular weight of 249.08, as well as Oxalic acid (HO_2_CCO_2_H) with a molecular weight of 90.03, were obtained from Sigma Aldrich Co. Elsewedy Electric Company supplied the XLPE material.

### Preparation of Co_3_O_4_ nanocomposite and *e-*beams irradiation process

A typical experiment created Co_3_O_4_ nanoparticles using the traditional sol–gel process. Solution A of the salt was created by dissolving 20.196 g (0.10 mol) of cobalt acetate in 600 mL of water/ethanol in a ratio 80/20 v/v% and stirring it for 60 min at room temperature. A solution of 0.20 mol of oxalic acid dehydrates was obtained by dissolving 2.520 g in 800 mL of water/ethanol in a ratio of 80/20 v/v% and stirred at a temperature of 50 °C for 60 min to create Solution B. Warm solution A was constantly mixed for one hour while solution B was added dropwise. A white sol was obtained, aged to create a gel, and then dried for 24 h at 100 °C. Co_3_O_4_ was finally produced using thermal processing at calcination temperatures of 600 °C. The obtained dried powder of Co_3_O_4_ was subsequently mixed with the appropriate amount of H_2_O_2_ (20%) for 120 min. After that, the mixture of (Co_3_O_4_ and H_2_O_2_) was dried in an oven at a temperature of 250 °C. After 3 h, the black precipitate of (Co_3_O_4_) was washed with de-ionized water and calcination at a muffle furnace at a temperature of 900 °C for 2 h before being subject to e-beam at doses of (0, 10, 20 and 30 kGy).

### Preparation of XLPE/Co_3_O_4_ by melt extruder method

The cross-linked polyethylene (XLPE) pellets were sourced from El Sewedy Electricity Company in Egypt, originally intended to manufacture high voltage (HV) cables with a rate 33 kV. The XLPE material was melted at 160 °C to initiate the composite fabrication process, utilizing a twin-screw extruder (CTW100P; Haake PolyLab RheoMix, Germany). Concurrently, Co_3_O_4_ nanoparticles, constituting 5 wt% and exposed to doses of 0, 10, 20, and 30 kGy, were introduced into the molten XLPE in its un-irradiated form and meticulously blended within the extruder. This amalgamation was achieved by maintaining a screw speed of 120 rpm for 7 min, ensuring thorough dispersion of the nanoparticles throughout the polymer matrix. After the extrusion stage, the composite mixture was subjected to compression-molding procedures utilizing a specialized hot press apparatus (Lab Tech Engineering Co., Bangkok, Thailand). During compression molding, the temperature was regulated at 170 °C, and a substantial pressure of 150 kg/cm^2^ was applied for 4 min. This rigorous molding process consolidated the XLPE/Co_3_O_4_ composite into a well-defined form. Upon completion of the molding procedure, the resultant XLPE/Co_3_O_4_ composite was meticulously sectioned into test specimens, each measuring 2 × 2 cm in dimensions. These prepared test pieces were then designated for subsequent rounds of comprehensive experimental assessments to elucidate and evaluate the material's multifaceted properties and behaviors, particularly in light of its exposure to different electron beam irradiation doses. The precise and controlled manufacturing protocol outlined above underscores the meticulous approach to creating the XLPE/Co_3_O_4_ composite specimens, ensuring uniformity and consistency in their composition and structure for subsequent scientific investigations and analyses.

### *e*-beams process

A linear particle accelerator (Vivi rad company, France) of 3.0 MeV electron flux with 30 mA at rated current and convyier speed 16 m/min was used to irradiate the samples. The dimension of source electron beam accelerator was (80 × 70) cm. The minimum dose was 3 kGy. The dose rate depended on convyier speed and current. The films were irradiated at ambient temperature by accelerated electrons until the absorbed doses from 10 to 30 kGy were reached.

### Measurements

The morphology of Co_3_O_4_ nanoparticles was examined by SEM EISS—EVO 15- UK scanning electron microscope at an accelerating voltage of 25 kV. Furthermore, the morphology and structures of the samples were investigated using transmission electron microscopy (TEM) with a JEOL-2010 instrument from Japan, providing detailed information about the nanoscale features and arrangement of the materials. XRD analysis was accomplished using SHIMADZU XRD 6000, operating at 1200 W, using the Cu-Kα radiation (λ = 1.5406 Å), 2θ ranges from 4 to 90. Unpaired electron analysis was achieved using BRUKER EMX EPR spectrometer whereas JASCO V-570 spectrophotometer was used to measure the diffused reflectance of Co_3_O_4_ nanoparticles and XLPE/Co_3_O_4_ nanocomposite in the spectral range from 190 to 2500 nm. For photoluminescence measurement, (SF-Jasco-FP-6500, Japan) spectrofluorometer was used and the samples were excited at 250 nm (laser diode) where the excitation slit band width was 5 nm. To explore the cationic oxidation states on the surface of Co_3_O_4_ nanoparticles, X-Ray photoelectron spectrometer (XPS) Thermo Scientific K-ALPHA instrument was used. The magnetic analysis was accomplished using Lake Shore 7410 Vibrating Sample Magnetometer (VSM). The dielectric measurements were carried out in the frequency range 10^–1^ to 10^7^ Hz at room temperature by employing Alpha-A machine from novocontrol. Samples were irradiated with 3 MeV electron beam at different dose (10, 20, and 30 kGy) at room temperature by Vivi rad (France) industrial linear electron accelerator in NCRRT, Egyptian Atomic Energy Authority. The conveyor speed is 16 m/min and the current was 30 mA.

### Quantification of physical parameters influenced by *e*-beam irradiation of Co_3_O_4_ nanoparticles and XLPE/Co_3_O_4_  nanocomposite

Electron beam (e-beam) irradiation has gained significant attention as a versatile technique for modifying the properties of materials at the nanoscale. In nanomaterials, cobalt oxide (Co_3_O_4_) is a promising compound with applications ranging from catalysis to energy storage. This study delves into the effects of e-beam irradiation on Co_3_O_4_ nanoparticles and XLPE/Co_3_O_4_ nanocomposite, aiming to quantify changes in various physical parameters.

#### X-ray diffraction (XRD) analysis

XRD analysis is a powerful technique for probing crystallographic information and quantifying various structural characteristics. The following equations were employed to derive essential parameters:

##### Crystallite size calculation

The crystallite size (D) was calculated utilizing Scherrer’s equation^53^:

1 $$D=\frac{\mathrm{K\lambda }}{\mathrm{\beta cos\theta }} ,$$

where D represents the crystallite size, K is a constant (K = 0.94), λ denotes the wavelength of X-rays used, θ corresponds to the Bragg's angle, and β signifies the full width at half maximum.

##### Unit cell length (a) calculation

The unit cell length (a) was determined based on the cubic structure of Co_3_O_4_ using the relation^54,55^:

2 $$\frac{1}{{d}^{2}}=\frac{{h}^{2}+{k}^{2}+{1}^{2}}{{a}^{2}},$$

Here, h, k, and l are the Miller indices associated with the crystal lattice.

##### Dislocation density calculation

The dislocation density (δ) was calculated using the formula^56,57^:

3 $$\delta =\frac{1}{{D}^{2}},$$

where δ represents the dislocation density and D is the crystallite size.

#### Optical band gap estimation

The optical band gap, a crucial parameter governing the material's light absorption behavior, was estimated using the Kubelka–Munk function. The equation utilized is as follows:

##### Kubelka–Munk function

The Kubelka–Munk function (F(R%)) was employed for optical band gap estimation^58^:

4 $${\text{F}}\left(\mathrm{R\%}\right)=\frac{{\left(1-R\%\right)}^{2}}{2R\%} ,$$

R% denotes the diffused reflectance, and F(R%) is the Kubelka–Munk function.

#### Impedance spectroscopic study

Impedance spectroscopy offers insights into the electrical properties of materials, particularly their conductivity. The following equation was used to quantify DC conductivity:

##### DC conductivity calculation

The DC conductivity (σ) was calculated using the equation^59–61^:

5 $$\upsigma =\frac{{\text{L}}}{{R}_{b}\times {\text{A}}},$$

where σ represents the DC conductivity, L denotes the membrane thickness, A signifies the area, and R_b_ is the bulk resistance.

The meticulous application of these equations enabled the determination of critical physical parameters, contributing to a comprehensive characterization of the materials under investigation. By quantifying these parameters, the study advances our understanding of the materials' structural, optical, and electrical properties, paving the way for potential applications and further scientific insights.

## Results and discussion

### The physiochemical properties of *e*-beam irradiated Co_3_O_4_ nanoparticles

The physicochemical properties of the electron beam (*e*-beam) irradiated Co_3_O_4_ nanoparticles refer to the changes and characteristics observed in the Co_3_O_4_ nanoparticles after they have been subjected to e-beam irradiation. This irradiation process can induce modifications in various aspects of the nanoparticles' structure and behavior.

#### SEM and TEM analysis

**Figure 1:**
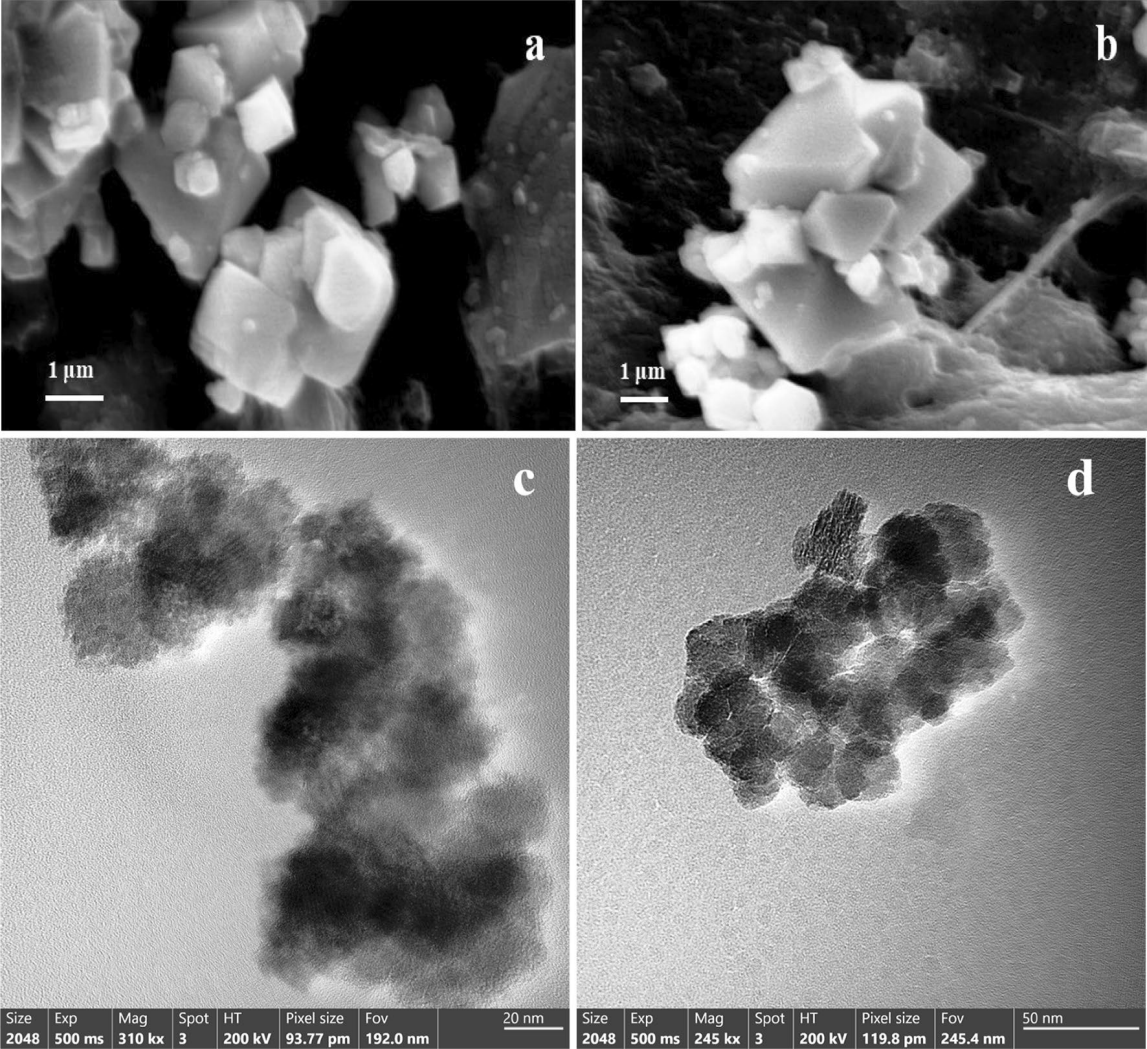
SEM images of the as prepared Co_3_O_4_ nanoparticles at two magnification levels where: (**a**) 10000x, (**b**) 8000x, and TEM images at: (**c**) 20 nm and (**d**) 50 nm levels.

Figure 1a shows SEM image of the as-prepared Co_3_O_4_ nanoparticles, mostly composed of truncated octahedrons (enclosed by [100] and [111] facets) with edge lengths ranging from 20 to 150 nm. The size of the Co_3_O_4_ cube particle is non-uniform, as depicted in Fig. 1a, which shows some irregular Co_3_O_4,_ including the grains that are recombined in the nanoparticles products are formed. In Fig.  1b we can see some truncated octahedrons agglomeration particles. There is almost coercivity attraction for Co_3_O_4_ nanoparticles, which is a very typical behavior for a soft magnet^62^. This intriguing behavior, often associated with materials possessing low coercivity values, underscores the unique magnetic properties of the Co_3_O_4_ nanoparticles within the system under study. The fact that the Co_3_O_4_ nanoparticles in the study exhibit a strong tendency to cluster together due to their mutual magnetic attraction suggests that these nanoparticles possess low coercivity. This low coercivity is a typical behavior of soft magnetic materials and is significant because it indicates the unique magnetic properties of the Co_3_O_4_ nanoparticles in the investigated system. Figure 1c and 1d show the TEM image of Co_3_O_4_ in the nanoscale with non-uniform cubic shape.

#### XRD analysis

**Figure 2:**
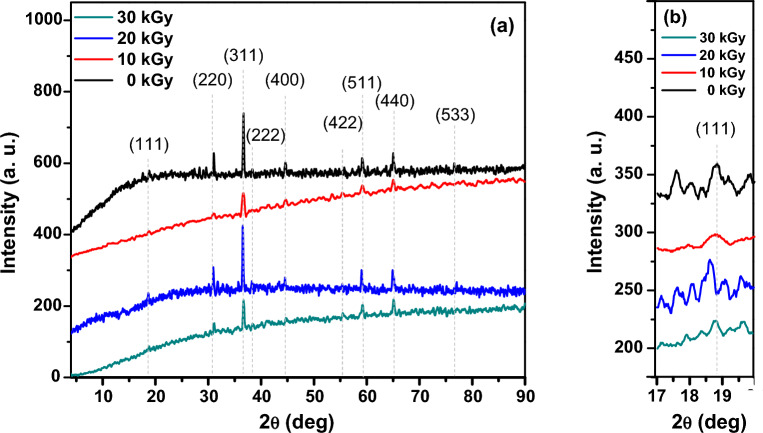
(**a**) The XRD patterns of Co_3_O_4_ before and after irradiation by different doses of electron beam and (**b**) the effect of radiation dose on the (111) plane growth.

Figure 2a shows a characteristic XRD pattern of the cubic phase of Co_3_O_4_ with a spinel structure. The XRD planes of cobalt oxide before irradiation appear at 2θ values 18.79º, 31.04º, 36.63º, 38.31º, 44.58º, 55.74º, 59.15º, 65.01º and 76.57º which are related to (111), (220), (311), (400), (511), (222), (440) and (533) diffraction planes of face-centered cubic spinel Co_3_O_4_, respectively. This is in agreement with JCPDS Card No. 42-1467. The observed peaks matching the Bragg reflections of the standard face-centered cubic (fcc) structure. No other phases, such as the hexagonal CoO wurzite is discernible. As the dose increases from 0 to 10 kGy and then 30 kGy, the crystallite size of the Co_3_O_4_ nanoparticles decreases from 47.55 to 18.50 nm and then 31.51 nm, respectively. This indicates a decrease in crystallinity with higher *e-*beams doses. However, at an intermediate dose of 20 kGy, the crystallite size increases to 51.26 nm, while the dislocation density decreases. This suggests that the nanoparticles exposed to 20 kGy have higher crystallinity compared to the residue Co_3_O_4_ nanoparticles. The highest dislocation density (2.921 × 10^15^ Lines/m^2^) is observed for the nanoparticles exposed to 10 kGy, indicating the most distorted crystal structure at this dose (Table 1). The lowest dislocation density (0.38 × 10^15^ Lines/m^2^) is observed for the nanoparticles exposed to 20 kGy, suggesting a more ordered crystal structure at this intermediate dose.

**Table 1:** XRD calculated parameters of Co_3_O_4_ nanoparticles before and after irradiation by different doses of electron beam.

Sample	D (nm)	a (Aº)	δ × 10^15^ (Lines/m^2^)
Co_3_O_4_ (0 kGy)	47.55	8.128	0.442
Co_3_O_4_ (10 kGy)	18.50	8.138	2.921
Co_3_O_4_ (20 kGy)	51.26	8.154	0.380
Co_3_O_4_ (30 kGy)	31.51	8.121	1

Figure 2b shows the XRD of cobalt oxide before and after irradiation by different e-beam doses (0, 10, 20, and 30 kGy). The peaks of [111] plane observed in the XRD patterns show little shift and different intensities correspond to specific crystallographic planes within the Co_3_O_4_ crystal lattice. These can reveal alterations in the arrangement of atoms within the crystal structure after exposure to *e-*beams. Figure 2b shows the changing of growth orientation-controlled (111) under the influence of *e*-beam. As observed, the *e*-beams doses cause the increase of (111) intensity with the shift in the position of 2θ to confirm the growth rate of (111) by *e*-beams irradiation. More interestingly, in Fig. 2a it is revealed that the increase of *e*-beams doses leads to alteration of the intensity of (311) plane as a preferred orientation of Co_3_O_4_ nanocrystals.

Furthermore, the decrease in crystal size for (311) plane was found with the growth in the crystal size for (111) plane. These findings provide an approach to the orientation-controllable Co_3_O_4_ nanocrystals of (111) plane as subjected to *e*-beams. This could be ascribed to the different spins of electrons which result in a redistribution of the density of states of the exposed nanoparticlesas a whole and can affect the magnetic properties of the Co_3_O_4_^63^. Since the catalytic reactions are affected by crystal facets according to their adsorption energies as well as the ability to transfer electrons, we followed the growth of (111) plane, which is entirely occupied by Co^2+^ ions and is reported to be responsible for the high catalytic performance of Co_3_O_4_ nanoparticles^64–66^. As shown in Fig. 2b, Co_3_O_4_ nanoparticles exposed to 20 kGy show the highest peak intensity of (111) plane whereas the Co_3_O_4_ nanoparticles exposed to 10 kGy show the lowest. Besides, the intensity of (111) plane of the irradiated Co_3_O_4_ nanoparticles at a dose of 30 kGy has been decreased compared to the un-irradiated Co_3_O_4_ nanoparticles. It is reported that while Co^3+^ ions change their positions, the Co^2+^ ions occupy the lattice point of (111) facet^67,68^ which is responsible for the magnetic behavior of Co_3_O_4_ nanoparticles^69^. Relating to the oxidation effect of electron beams^70,71^, some of Co^2+^ ions on the surface could be converted to Co^3+^ ions, consequently affecting the magnetic behavior of Co_3_O_4_ nanoparticles. It is also reported that the different crystal facets possess different adsorption energies and electron transfer properties, which might directly influence the catalytic reaction and ionic conductivity of Co_3_O_4_ nanoparticles^65^. So, the XRD results suggest that the orientation-controlled (111) Co_3_O_4_ nanocrystals could change the materials' magnetic and electrical properties.

**Optical** **analysis** **for** **Co**_**3**_**O**_**4**_ **nanoparticles**

The change in the absorption and the emission properties and the variation in the band gap of the nanoparticles may be due to the quantum confinement effect associated by alteration of crystallite size and shape as well as defects induced by radiation that could initiate localized states which affect the absorption as well as emission characteristics of the nanoparticles^72^.

##### Optical band gap

**Figure 3:**
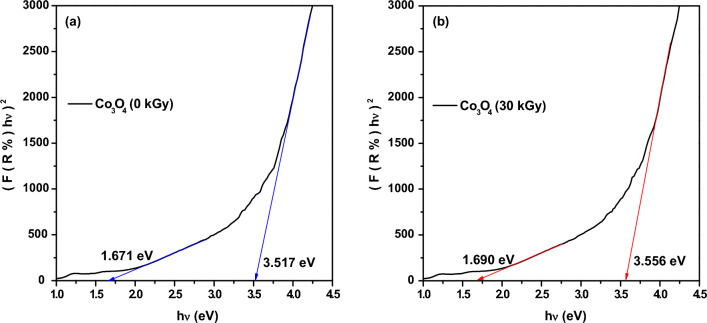
Diffused reflectance spectra of Co_3_O_4_ nanoparticles versus hυ using Kubelka–Munk function: (**a**) before and (**b**) after irradiating by electron beam radiation at a dose of 30 kGy.

The optical band gap (E_g_) of Co_3_O_4_ nanoparticles (before and after irradiation by 30 kGy of electron beam) has been estimated from the diffused reflectance spectra using Kubelka–Munk function (Eq. 4). By plotting (*F*(*R*) hυ)^2^ versus hυ and extrapolating the linear part of the curve to *F*(*R*) = 0, the optical band gap value of the samples could be determined. As revealed from Fig. 3, Co_3_O_4_ nanoparticles show two direct optical transitions before and after electron beam irradiation. This could be attributed to its electronic band structure; As cobalt ion has two oxidation states (Co^3+^ and Co^2+^) there would be two associated charge transitions (O^2-^ to Co^3+^ and O^2–^ to Co^2+^) which are responsible for the lower and higher optical absorption bands, respectively. For the un-irradiated Co_3_O_4_, the lower one is at 1.671 eV whereas the higher one is at 3.517 eV. These values agree well with previous reports^72–75^. However, these values have been increased to 1.690 eV and 3.556 eV, respectively as Co_3_O_4_ nanoparticles have been irradiated by electron beam at a dose of 30 kGy. This blue shift could be related to the decreased crystallite size upon irradiation by electron beam which agrees with XRD results. Besides, the quantum confinement effect could also cause this increase in the optical band gap. Additionally, the oxidation effect of electron beam could also be another cause of increasing the band gap as some of Co^2+^ would be converted to Co^3+^ and change their position to the octahedral cites instead of occupying the tetrahedral cites which could perturb the electronic structure of the spinel Co_3_O_4_ nanoparticles.

##### Photoluminescence analysis

The photoluminescence analysis (PL) is inspected to analyze the optical emission properties of Co_3_O_4_ nanostructures and to confirm the presence of oxygen vacancies in the un-irradiated Co_3_O_4_ nanoparticles and to compare the spectrum with that of the irradiated Co_3_O_4_ nanoparticles at a dose of 30 kGy of electron beams.

**Figure 4:**
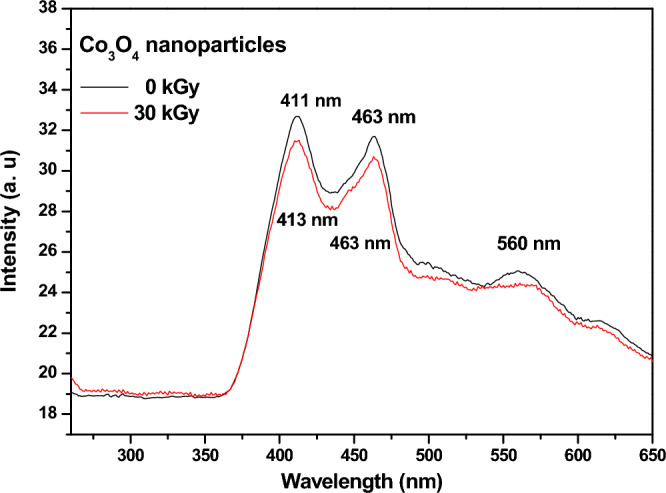
A comparison between photoluminescence spectra for Co_3_O_4_ nanoparticles before and after irradiation at a dose of 30 kGy of electron beams.

When excited at 250 nm (λ_excitation_), the emission spectrum of un-irradiated Co_3_O_4_ nanoparticles show a broad peak that has two maxima (Fig. 4), the deeper emission originates at 411 nm which refers to the blue emission whereas the other maximum is at 463 nm which denotes the green emission. The shallow peak that appears at 560 nm is also in the green emission range and reflects the presence of defects and oxygen vacancies within the nanoparticles which is responsible for the stabilization of Co_3_O_4_ cubic spinel structure, in agreement with previous reports. It is worth noting that blue band is ascribed to the O^2−^–Co^2+^ charge-transfer process whereas the green emission is related to the O^2−^– Co^3+^ charge transfer^76^. The deep peak in the blue range and the weak green emission confirm the good crystallinity of our prepared nanoparticles^77^.

Upon irradiating the samples at a 30 kGy of electron beams, no significant shift on the two main peaks of Co_3_O_4_ nanoparticles has been observed. However, the shallow peak (at 560 nm) which denotes the presence of the oxygen vacancies has been damped which is suitably related to the oxidation effect of electron beams on the surface Co^2+^ ions.

#### XPS analysis

**Figure 5:**
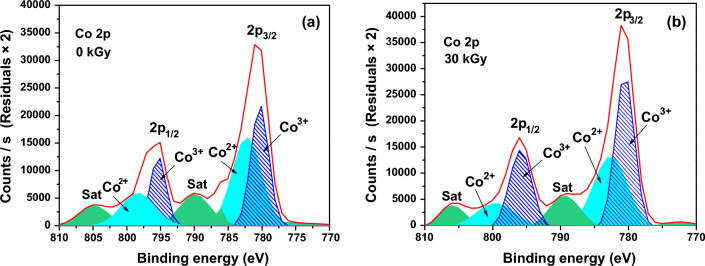
XPS spectra of Co 2p of the Co_3_O_4_ nanoparticles: (**a**) before and (**b**) after electron beam irradiation at a dose of 30 kGy.

To further investigate the alterations on the oxidation states of cobalt ions on the surface of Co_3_O_4_ nanoparticles upon irradiation, X-ray photoelectron spectroscopy (XPS) was carried out. As shown in Fig. 5a, the Co 2p spectrum of Co_3_O_4_ nanoparticles before irradiation indicates a low energy and a high energy band at 781.08 eV and 795.08 eV, corresponding to Co 2p_3/2_ and Co 2p_1/2_, with two satellites at 790.08 eV and 805.08 eV, and these are the characteristic of Co_3_O_4_ phases^78^. The Co 2p_3/2_ peak of 781.08 eV can be further disassembled into two fitting peaks at 780.08 eV and 782.08 eV, and the Co 2p_1/2_ peak of 795.08 eV can be further disassembled into two fitting peaks at 795.08 eV and 798.08 eV. The peaks with the binding energy of 780.08 eV and 795.08 eV can be appointed to Co^3+^, while the peaks at 782.08 eV and 798.08 eV can be appointed to Co^2+^.

It is observed from Fig. 5b that upon irradiating the Co_3_O_4_ nanoparticles at a dose of 30 kGy of electron beam, a slight shift of the peaks corresponding to Co^2+^ to higher binding energies. Additionally, the area ratio of surface Co^2+^ against Co^3+^ has decreased from 52.88% to 42.28% which infers that Co^3+^ has become the predominant on the surface and some of Co^2+^ has transformed to Co^3+^ as a result of exposure to electron beam radiation. This could be ascribed to the oxidation effect of the ionizing radiation reported by many researchers, and consequently, some surface oxygen vacancies vanishing. This agrees well with the photoluminescence result which refers to fewer oxygen vacancies on the surface as Co_3_O_4_ nanoparticles has been irradiated at a dose of 30 kGy of electron beam.

#### ESR and magnetic analysis

**Figure 6:**
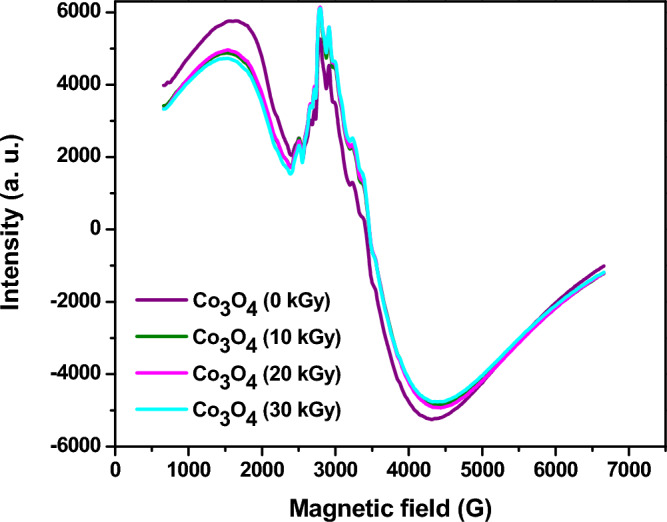
ESR spectra of Co_3_O_4_ nanoparticles before and after exposure to different doses of electron beam.

The ESR spectra of Co_3_O_4_ nanoparticles are investigated at room temperature (300 K) before and after exposure to e-beam at different doses (10, 20, and 30 kGy) as illustrated in Fig. 6. The ESR signal of un-irradiated Co_3_O_4_ is centered at about 3400 G which is attributed to Co^2+^ ion that have 3d7 electronic configuration. The isotropic g-factor (g) representing the spectroscopic splitting factor and measures the electronic environment around the paramagnetic Co^2+^ ions within the nanoparticles, remains relatively constant around 2.048 before and after irradiation at a dose of 10 kGy. This indicates little change in the electronic environment at the lowest subjected irradiation dose. At irradiation doses of 20 and 30 kGy, the g-factor increases slightly. This suggests that electron beam exposure begins to perturb the electronic environment around the Co^2+^ ions, resulting in a higher g-factor. This is a slight shift of the resonance field to higher magnetic fields upon irradiation. The Δg value, representing the deviation from the free electron g-factor of 2.0023, also increases slightly with higher irradiation doses, confirming the changes to the electronic structure induced by electron beam exposure. Also, its negative sign infers the ionic bonding in Co_3_O_4_ nanoparticles^79^. The ESR signal intensity, proportional to the number of unpaired electrons, also increases with rising irradiation dose. This indicates that electron beam irradiation generates more free radical species within the nanoparticles. The partial elimination of oxygen vacancies due to exposure to e-beam radiation and freeing of some trapped electrons could be the reason for this increase in the signal intensity^80^. The increase in g-factor, Δg and ESR intensity with higher irradiation doses suggests that electron beam exposure slightly distorts and defects the crystal structure of Co_3_O_4_, generating more free electrons and paramagnetic centers. It is also observable that the radiation dose has nearly no effect on the signal shape or size which infers that the oxygen vacancies only initiates on the surface so it quickly vanishes upon irradiation and the alteration only appears in the signal intensity.

Table 2 shows trends in the isotropic factor (g), g-shift (Δg) and ESR signal intensity of Co_3_O_4_ nanoparticles after exposure to different doses of electron beam irradiation. The isotropic factor g remains relatively constant around 2.048, indicating that the electron spin state of the Co_3_O_4_ nanoparticles is not significantly altered by electron beam exposure up to 30 kGy. The g-shift (Δg) shows a small increase with irradiation dose, suggesting a slight change in the electron spin configuration upon electron beam exposure. However, the absolute values of the g-shift remain very small. Most notably, the ESR signal intensity, proportional to the number of unpaired electrons, increases dramatically with electron beam dose. There is a 6% increase in signal intensity from the unirradiated nanoparticles to those exposed to 30 kGy. This data suggests a significant increase in unpaired electrons detected by ESR spectroscopy. This could be due to vanishing oxygen vacancies on the surface of nanoparticles upon irradiation, freeing traped unpaired electrons and, thus a stronger ESR signal.

**Table 2:** The isotropic factor, g and signal intensity of Co_3_O_4_ nanoparticles at different doses of electron beam.

Dose (kGy)	g	Δg	Signal intensity (counts/g)
0	2.04807	− 0.04577	99,790.87
10	2.04807	− 0.04577	100,388.17
20	2.04809	− 0.04579	102,954.33
30	2.04818	− 0.04588	106,197.45

In summary, electron beam irradiation appears to generate more electron spin centers within Co_3_O_4_ nanoparticles, as evidenced by the increase in ESR signal intensity with dose, while having little effect on the actual electron spin state, as indicated by the relatively constant g values. The enhanced number of unpaired electrons could influence the magnetic and catalytic properties of the irradiated nanoparticles. This suggests irradiation induces crystallographic distortions, eliminates oxygen vacancies that generate more paramagnetic centers within the Co_3_O_4_ nanoparticles.

**Figure 7:**
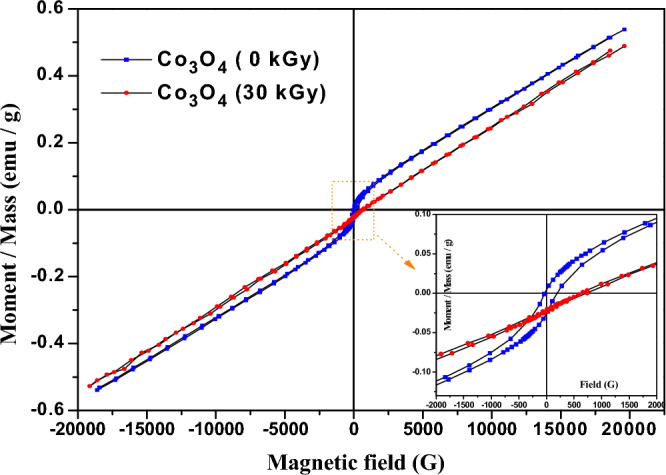
The hysteresis curve of Co_3_O_4_ nanoparticles before and after irradiation by electron beam at a dose of 30 kGy.

The magnetization measured at room temperature as a function of the applied field (hysteresis loop) for Co_3_O_4_ nanoparticles before and after exposure to 30 kGy of electron beams is illustrated in Fig. 7. Our investigated Co_3_O_4_ nanoparticles are characterized by three important parameters; the coercivity (H_c_) which represents the strength of the applied magnetic field needed to reduce the nanoparticles' magnetization to zero after saturation, the remanent magnetization (M_r_) which refers to the magnetic moment that the nanoparticles retain without an external magnetic field where the non-zero M_r_ also indicates ferromagnetic behavior, and the saturation magnetization (M_s_) which refers to the maximum magnetic moment attained by the nanoparticles when an external magnetic field is applied. It is observed that Co_3_O_4_ nanoparticles revealed a weak ferromagnetic behavior in which the coercive field (H_c_) is 112.57 G, the remanent magnetization (M_r_) is 15.076E-3 emu/ g, and the saturation magnetization (M_s_) is 0.538 emu/g. The saturation magnetization (which characterizes the ferromagnetic behavior) of previously reported works compared to our work is found in Table 3. Many researchers have reported the ferromagnetic behavior of the nanosized Co_3_O_4_ particles in converse to its bulk behavior (anti-ferromagnetic)^81,82^. In nanosized materials, the existence of oxygen vacancies which mainly establish on the particles surface is responsible for trapping unpaired electrons, generating ferromagnetic order as their spins will polarize together^83^. Besides, the exchange interaction between Co^2+^ and Co^3+^ ions at the tetrahedral and octahedral sites in spinel Co_3_O_4_ and the density of states redistribution can influence the magnetic properties of the Co_3_O_4_^63^.

**Table 3:** Saturation magnetization of Co_3_O_4_ nanoparticles prepared at different crystallite sizes by different preparation methods and conditions compared to our result.

Preparation method	Size (nm)	M_s_ (emu/g)	References
Co-precipitation method	58	0.813	^84^
Precipitation method	36	0.362	^85^
Thermal decomposition at 175 ºC	11	0.137	^86^
Microwave combustion method	45.8	0.005	^87^
Sol–gel method	47.55	0.538	Present work

It is also reported that the magnetic properties of Co_3_O_4_ is affected by its crystal field. In an octahedral field, the five degenerate d orbitals of Co ions split into two sets of orbitals (t_2g_ and e_g_) where t_2g_ are three low energy orbitals whereas e_g_ are two high energy orbitals. The difference between these sets of orbitals depends on the ligand nature; increases with strong ligands. In the presence of strong ligand, the lower orbitals are filled before the higher orbitals and the Co ion will be in low-spin state whereas in the presence of weak field ligand, the electrons fill the orbitals in a Hund’s rule type of order and pair up only when they have no other choice and the Co ion will be in a high spin state. In case of a tetrahedral field, the energy levels are reversed and the d orbitals split into two lower lying fully occupied e - states and three half-filled t_2_ - states^88^, respectively where the difference between these sets of orbitals is nearly half that in case of the octahedral field^89^ so, the electrons fill the orbitals in a high spin state regardless of the ligand nature. In the spinel structured Co_3_O_4_ nanoparticles, the surrounding O ions represent a strong ligand so the magnetic configuration of Co^3+^ that occupy the octahedral sites^90,91^ is S = 0 (t_2g_^6^ e_g_^0^).

In contrast, the magnetic configuration of Co^2+^ that normally occupy the tetrahedral sites is S = 3/2 (e_g_^4^ t_2g_^3^), which confirms that the antiferromagnetic behavior of Co_3_O_4_ in the bulk case comes from the Co^2+^ ions as a result of their three unpaired electrons^69^. In the nanoscale, as in our system, the presence of magnetic moment is largely related to the oxygen vacancies as well as the crystal symmetry deficiency, since Co^3+^ ions have no magnetic moment^90^ and Co^2+^ cancels its total magnetic moment due to its antiferromagnetic nature. However, the observed weak ferromagnetic nature of our system may be related to surface uncompensated Co^2+^ and/or the surface oxygen vacancies that mainly arise in the nanoscale system as a result of the weak coordination on the surface as well as the loss of translational symmetry^83^.

However, the irradiated Co_3_O_4_ nanoparticles (30 kGy) show lower values of these magnetic parameters than those of the un-irradiated Co_3_O_4_ nanoparticles as shown in Table 4. This could be attributed to oxidation and freeing of some trapped electrons due to irradiation. This behavior agrees with XPS and PL analyses that concluded the decrease of Co^2+^ ions (responsible for unpaired electrons) and oxygen vacancies on the surface upon irradiation, respectively.

**Table 4:** Magnetic parameters of Co_3_O_4_ nanoparticles and XLPE/Co_3_O_4_ nanocomposite before and after irradiation.

Sample	H_c_ (G)	M_r_ (emu/g)	M_s_ (emu/g)	Total area (erg/g)	Squareness (Mr/Ms)
Co_3_O_4_ (0 kGy)	112.57	15.076E–3	0.538	165.780	27.988E–3
Irradiated Co_3_O_4_ (30 kGy)	49.51	1.7408E–3	0.508	34.590	3.4254E–3
XLPE/Co_3_O_4_	175.72	3.9082E–3	72.445E-3	61.470	53.948E–3
Irradiated XLPE/Co_3_O_4_ (30 kGy)	135.18	857.93E–6	23.644E-3	16.423	36.286E–3

### The physiochemical properties of irradiated (XLPE/Co_3_O_4_) nanocomposite

As the magnetic and electrical properties of Co_3_O_4_ nanoparticles could be enhanced by the presence of oxygen vacancies^38,92^ so we have chosen the un-irradiated Co_3_O_4_ nanoparticles, as having the highest oxygen vacancies, to be filled in XLPE and to investigate the structural, optical, magnetic, and electrical properties of the resultant nanocomposite before and after irradiation by electron beams.

#### XRD

**Figure 8:**
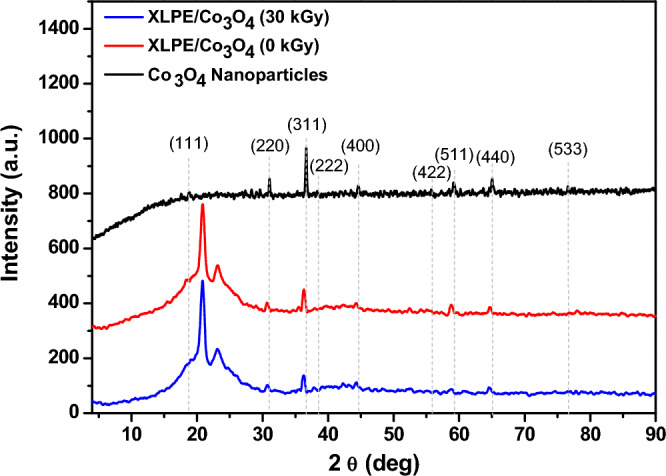
The XRD patterns of XLPE/Co_3_O_4_ nanocomposite before and after irradiation by 30 kGy of electron beam compared to that of the as prepared Co_3_O_4_ nanoparticles.

Upon filling the un-irradiated Co_3_O_4_ nanoparticles in XLPE matrix, the same lattice planes of Co_3_O_4_ nanoparticles appear with a slight shift from their positions and the peaks concerning XLPE appear around 2θ = 20º (Fig. 8). The XRD pattern of XLPE/Co_3_O_4_ nanocomposite after irradiation by 30 kGy of the electron beam is also depicted in Fig. 8. The lattice parameters of Co_3_O_4_ nanoparticles have been calculated as altered after embedding in XLPE matrix. The calculated values are shown in Table 5 for comparison.

**Table 5:** XRD calculated parameters of XLPE/Co_3_O_4_ before and after irradiation at a dose of 30 kGy of electron beam compared to those calculated for un-irradiated Co_3_O_4_ nanoparticles.

Sample	D (nm)	a (Aº)	δ × 10^15^ (Lines/m^2^)
Co_3_O_4_ (0 kGy)	47.55	8.128	0.442
XLPE/ Co_3_O_4_ (0 kGy)	18.49	8.203	2.925
XLPE/ Co_3_O_4_ (30 kGy)	18.91	8.216	2.793

From Table 5**,** the Co_3_O_4_ nanoparticles (0 kGy) have a crystallite size of 47.55 nm and a lattice constant of 8.128 A^o^. After being embedded in the XLPE matrix, the crystallite size decreases significantly to 18.49 nm. This suggests that the XLPE matrix restricts the growth of the Co_3_O_4_ nanoparticles. However, the lattice constant increases after embedding in XLPE, indicating some strain induced by the polymer matrix on the nanoparticles. The dislocation density also increases markedly after the Co_3_O_4_ nanoparticles are embedded in XLPE, from 0.442 × 10^15^ to 2.925 × 10^15^ Lines/m^2^. This suggests that the incorporation process induces defects and distortions in the nanoparticles. After irradiation of the XLPE/Co_3_O_4_ composite with 30 kGy of electron beam, the crystallite size and lattice constant increase slightly to 18.91 nm and 8.216 Aº, respectively whereas the dislocation density decreased marginally after 30 kGy irradiation. This could be due to some restructuring (enhanced crystallinity) and growth of the nanoparticles under irradiation. These slight changes in the XRD parameters indicate that the crystal structure of the Co_3_O_4_ nanoparticles remains relatively stable within the XLPE matrix upon moderate electron beam exposure.

#### Optical band gap

**Figure 9:**
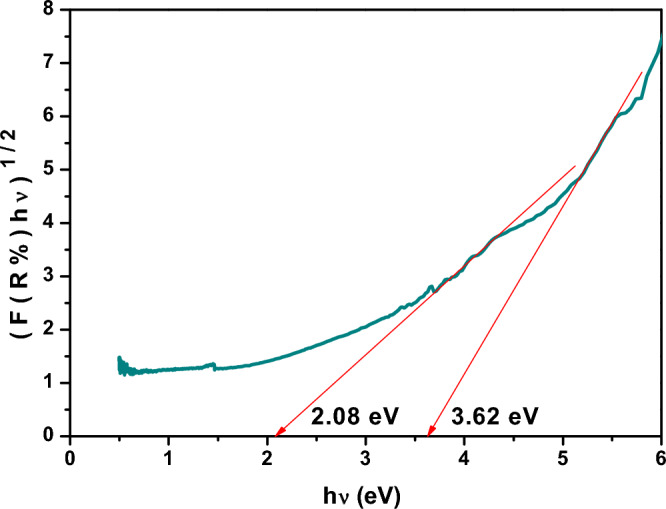
Diffused reflectance spectra of neat XLPE versus hυ using Kubelka–Munk function.

**Figure 10:**
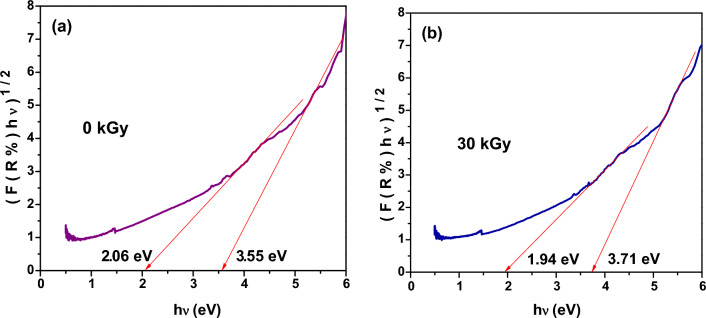
Diffused reflectance spectra of XLPE/Co_3_O_4_ nanocomposite versus hυ using Kubelka–Munk function: (**a**) before and (**b**) after exposure to electron beam radiation at a dose of 30 kGy.

The optical band gap (E_g_) of un-irradiated XLPE and XLPE/Co_3_O_4_ nanocomposite (before and after irradiation by electron beam) has also been estimated from the diffused reflectance spectra using Kubelka–Munk function (Eq. 4). By plotting (*F*(*R*) hυ) ^1/2^ versus hυ and extrapolating the linear part of the curve to *F*(*R*) = 0, the optical band gap value of the samples could be determined as shown in Fig. 9 and 10. It is observed that XLPE (Fig. 9) shows two indirect optical transitions, the lower one is at 2.08 eV whereas the higher one is at 3.62 eV.

However, upon filling XLPE with Co_3_O_4_ (Fig. 10), the lower gap slightly decreased to 2.06 eV whereas the higher gap increased to 3.55 eV. This slight shift as Co_3_O_4_ filled XLPE matrix could be ascribed to the low weight percent (5%) of Co_3_O_4_ nanoparticles which have also two energy gaps which are relatively lower than these values, the lower one is at 1.671 eV whereas the higher one is at 3.517 eV. It is important to note that the presence of Co_3_O_4_ nanoparticles introduces additional energy states that can contribute to the observed energy transitions in the nanocomposite. However, upon irradiating XLPE/Co_3_O_4_ nanocomposite at a dose of 30 kGy of electron beam (Fig. 10), a red shift from these values has been achieved in the lower value which could be attributed to cross linking and imperfections induced by radiation which could decrease the optical band gap^93,94^. However, the higher band gap value of the nanocomposite exposed to 30 kGy exceeds that of the un-irradiated one. The spinel structure of Co_3_O_4_ with Co^2+^ and Co^3+^ ions distributed across octahedral sites which is responsible for the two different band gaps could be affected by electron beam induced oxidation which consequently would affect both the optical band gaps and the electronic conductivity of the polymeric nanocomposite. The estimated values of the low and high optical band gaps of XLPE and XLPE/Co_3_O_4_ nanocomposite before and after irradiation at a dose of 30 kGy of electron beam are listed in Table 6 for comparison.

**Table 6:** Optical band gap values of XLPE/ Co_3_O_4_ nanocomposite before and after exposure to electron beam radiation at a dose of 30 kGy.

Sample	E_g1_ (eV)	E_g2_ (eV)
XLPE (0 kGy)	2.08	3.62
XLPE/Co_3_O_4_ (0 kGy)	2.06	3.55
XLPE/Co_3_O_4_ (30 kGy)	1.94	3.71

#### ESR and magnetic analysis

**Figure 11:**
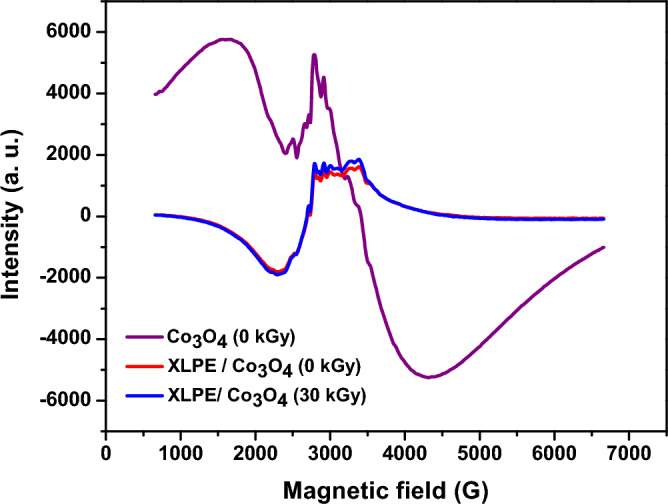
ESR spectra of XLPE/Co_3_O_4_ nanocomposites before and after electron beam irradiating at a dose of 30 kGy compared to the spectrum of un-irradiated Co_3_O_4_ nanoparticles.

In investigating the ESR results of XLPE/Co_3_O_4_ nanoparticles, it is observed that the signal related to Co^2+^ ions has disappeared upon filling inside the polymeric matrix (Fig. 11). This could be attributed to the low weight fraction of the filler (~ 5%) as well as the restriction of surface electrons as embedded in the polymeric XLPE Matrix. It is worth noting that also no signal is observed after irradiating XLPE/Co_3_O_4_ nanocomposite with 30 kGy of electron beam.

**Figure 12:**
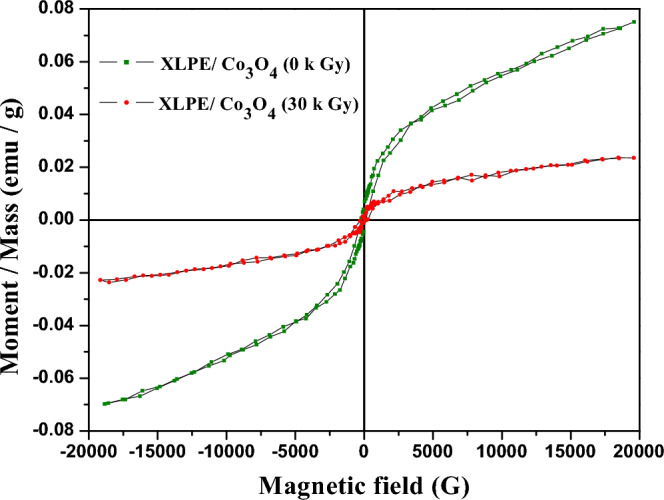
Magnetization versus applied field of XLPE/Co_3_O_4_ nanocomposite before and after 30 kGy electron beam exposure.

In investigating the magnetization versus magnetic field behavior of XLPE/ Co_3_O_4_ nanocomposites (Fig. 12), we observe that the nanocomposite has also gained a ferromagnetic behavior but with higher coercive field (H_c_) ~ 175.72 G than that of the un-irradiated Co_3_O_4_ nanoparticles. However, the remanent magnetization (M_r_), and saturation magnetization (M_s_) are reduced (see Table 4). The nanocomposite's increased coercivity and decreased retentivity may be caused by the polymer chains that restrict the easy response of electrons spin as the sample is magnetized and demagnetized. Additionally, the highly increased dislocation lines density (see Table 5) initiated during the polymerization process could cause domain wall pinning^47^. So, more external field would be exerted to unpin the domain wall from its pinned position and accordingly, the polymeric nanocomposite shows a greater coercivity. As expected, the irradiated nanocomposite (30 kGy) shows lower values of these magnetic parameters related to the oxidation effect of electron beam and the decrease in oxygen vacancies that trap the unpaired electrons. Besides, decreasing dislocation lines density upon irradiating the XLPE/Co_3_O_4_ nanocomposite at a dose of 30 kGy is also responsible for the decreased coercivity compared to the un-irradiated nancomposite. Also, the smaller area of the nanocomposite hysteresis loop before and after irradiation could be attributed to the low ratio of Co_3_O_4_ nanoparticles^46^ (~ 5%) in the XLPE matrix. The room temperature ferromagnetic behavior with relatively low values of retentivity and coercivity of our prepared nanocomposite before and after irradiation suggests using it as soft magnets which find applications in spintronics.

#### Dielectric analysis and complex conductivity of XLPE/Co_3_O_4_ nanocomposite

**Figure 13:**
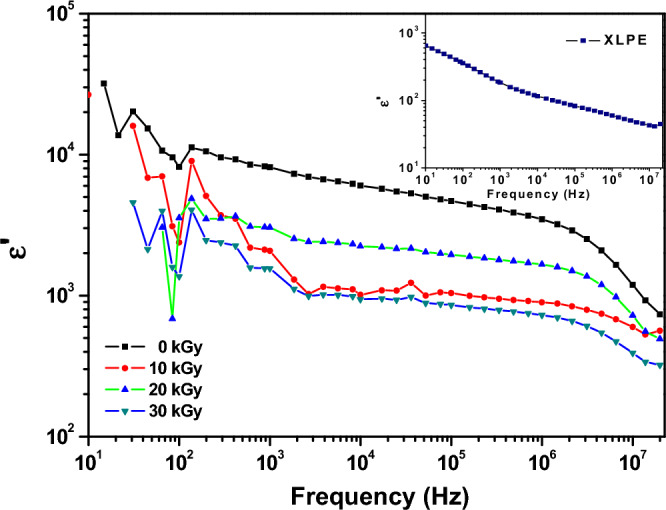
Frequency dependence of real dielectric permittivity ε′ of XLPE/Co_3_O_4_ nanocomposite exposed to different doses of electron beam. The inset shows the dielectric permittivity spectrum of neat XLPE.

Figure 13 show the effect of frequency, ranging from 10 to 10^7^ Hz at room temperature, on the dielectric permittivity of XLPE/Co_3_O_4_ nanocomposite before and after irradiation at 0, 10, 20, and 30 kGy of an electron beam. The high dielectric permittivity values (ε′) over all range of frequency are due to the semiconducting nature of the Co_3_O_4_ nanocomposites. These high values have been relatively decreased upon irradiation and the sample exposed to 30 kGy of electron beam shows the least values. Upon exposure to electron beam irradiation, the dielectric permittivity generally decreases. This trend is observed at all frequencies and for all irradiation doses. The sample irradiated with the highest dose of 30 kGy shows the lowest permittivity values over the entire frequency range. The reduction in permittivity upon irradiation suggests that radiation causes defects within the Co_3_O_4_ nanoparticles and the XLPE polymer matrix. These defects reduce the material's ability to store charge, lowering the dielectric constant. As the irradiation dose increases, more defects are generated, resulting in a further decrease in dielectric permittivity. The 30 kGy sample has accumulated the most defects and thus exhibits the lowest permittivity. The decrease in permittivity with increasing frequency can be attributed to space charge polarization and interfacial polarization effects, which become less prominent at higher frequencies. The electron beam irradiation induces defects within the XLPE/Co_3_O_4_ nanocomposite that reduce its ability to store charge, decreasing dielectric permittivity. Higher irradiation doses generate more defects and cause a further reduction in permittivity.

**Figure 14:**
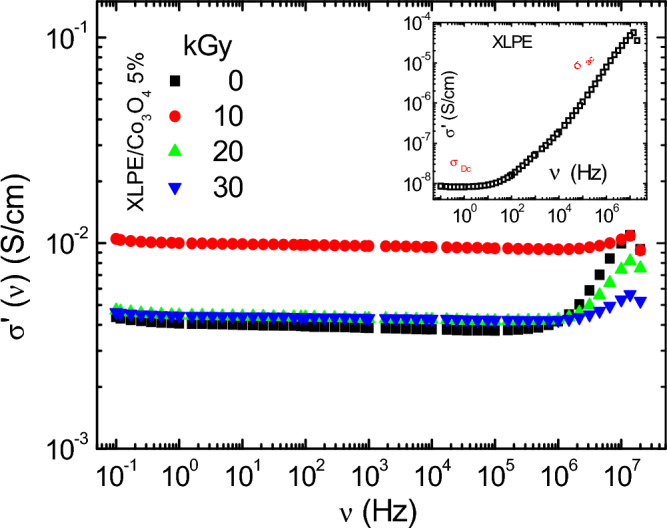
The real part of the conductivity σ′ (υ) as a function of frequency at room temperature for XLPE/Co_3_O_4_ nanocomposites exposed to 0, 10, 20, 30 kGy. The σ′ (υ) of neat XLPE film is in the inset.

Figure 14 portrays the frequency-dependent behavior of the real part of complex conductivity (σ') for the XLPE/Co_3_O_4_ nanocomposite subjected to varying electron beam irradiation doses (0, 10, 20, and 30 kGy). To provide context, the inset includes the real conductivity of neat XLPE polymer. A discernible transition in conductivity is evident, shifting from a power-law plateau characteristic (σ_DC_) to another state contingent on frequency (σ_AC_). This transition, marked by an inflection point, characterizes the response typical of disordered materials, particularly polymers^95^.

The σ_DC_ for the unirradiated XLPE/Co_3_O_4_ sample spans the range of 10^–3^ S/cm < σ < 10^–2^ S/cm, indicative of semiconductor behavior. This relatively higher DC conductivity is attributed to the ionic conductivity of Co_3_O_4_ nanoparticles, rather than electronic conductivity, given the slight reduction in the XLPE's optical band gap upon Co_3_O_4_ nanoparticle incorporation. Notably, the (111) planes within the Co_3_O_4_ spinel structure facilitate rapid diffusion paths for charged species, further enhancing ionic conductivity. Increased (111) plane population augments ionic conductivity potential.

Upon irradiation, a subtle rise in both AC and DC conductivity manifests. This phenomenon underscores irradiation's role in liberating charge carriers, enhancing their mobility across XLPE segments rather than inducing changes in electronic structure^96^. In polymers like XLPE, charge transport primarily involves hopping charge carriers (electrons or holes) between molecular chains. Electron beam irradiation incites bond breakage and defects within XLPE chains, resulting in free electrons and holes as mobile charge carriers. These carriers achieve heightened mobility within polymer segments, no longer restricted to individual molecules. They navigate between defect states created by irradiation, albeit modestly, bolstering AC and DC conductivity.

The abundance of (111) planes with their high atomic density and favorable atomic arrangement engenders optimal diffusion pathways for ions, enhancing ionic conductivity. The hexagonal arrangement within (111) planes facilitates bi-dimensional ion movement, while their low surface energy and stability accommodate ion migration with minimal distortion. This arrangement also entails lower activation energies for ion diffusion. The increased AC conductivity corresponds to the responsiveness of free charge carriers to alternating electric fields at higher frequencies, while the slight DC conductivity rise stems from additional charge carriers contributing to consistent current flow.

In sum, electron beam irradiation of the XLPE/Co_3_O_4_ nanocomposite induces defects that promote charge carrier mobility, resulting in a modest increase in AC and DC conductivity. The proliferation of (111) planes enhances ionic conductivity, facilitating charge carrier diffusion and reducing activation barriers. While the overall effect is relatively modest, this irradiation-induced conductivity enhancement holds significant potential for diverse applications necessitating improved charge transport and material performance**.**

#### Impedance spectroscopic study of XLPE/Co_3_O_4_ nanocomposite

**Figure 15:**
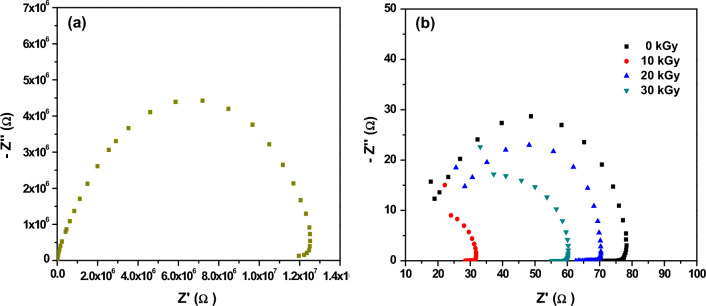
Nyquist impedance plot for (**a**) neat XLPE and (**b**) XLPE/Co_3_O_4_ nanocomposite at different doses of electron beam.

The Nyquist impedance plots at room temperature (RT) for neat XLPE and XLPE/Co_3_O_4_ nanocomposites before and after exposure to varying electron beam doses are depicted in Fig. 15a and 15b, respectively. All samples exhibit semi-circular patterns intersecting the X-axis, indicative of impedance predominantly influenced by the material's bulk resistance (R_b_) and capacitance (C). This characteristic signifies the contribution of bulk properties rather than solely surface effects.

The intercepts on the real axis at the lower frequency range denote the specimens' bulk resistance (R_b_), where lower R_b_ corresponds to higher conductivity^97,98^. The [111] planes within Co_3_O_4_ host an elevated density of under coordinated cobalt atoms, serving as active sites for electron conduction. The presence of Co_3_O_4_ nanoparticles shifts the semicircles towards higher frequencies, indicating diminished R_b_ and augmented conductivity compared to neat XLPE. The conductivity of the polymer nanocomposites was calculated through Eq. (5) from the complex impedance spectra (Fig. 15) and the calculated values are listed in Table 7.

**Table 7:** The conductivity values of un-irradiated XLPE and XLPE/Co_3_O_4_ composites before and after irradiation, as calculated from the impedance spectra.

Sample	σ (S/cm)
XLPE (0 kGy)	0.133 × 10^–7^
XLPE/Co_3_O_4_ (0 kGy)	2.198 × 10^–3^
XLPE/Co_3_O_4_ (10 kGy)	6.258 × 10^–3^
XLPE/Co_3_O_4_ (20 kGy)	3.262 × 10^–3^
XLPE/Co_3_O_4_ (30 kGy)	3.614 × 10^–3^

As depicted in Table 7 and Fig. 15, the DC conductivity has increased upon filling XLPE by Co_3_O_4_ nanoparticles; approximately multiplied by the fourth power of ten. However, a slight increase in DC conductivity is observed upon irradiation. Owing to the high ionic conductivity of transition metal oxides^99^, filling XLPE with Co_3_O_4_ nanofiller would cause a reasonable overall ionic conductivity of the polymer nanocomposite which could be applied as a good solid electrolyte. It is worth noting that XLPE/Co_3_O_4_ (10 kGy) possessed the higher Dc conductivity (6.258 × 10^–3^ S/cm).

Significantly, the [111] planes of Co_3_O_4_ play a pivotal role in ameliorating conductivity. Exposure to electron beam radiation further shifts the semicircles to higher frequencies, underscoring a more pronounced reduction in R_b_ and an intensified enhancement in conductivity. This is attributed to the ability of active sites on the [111] planes of Co_3_O_4_ to trap the additional electrons and holes produced by radiation, thereby facilitating charge transport.

The combined effect of Co_3_O_4_ nanoparticles and electron beam radiation synergistically contributes to the amplified DC conductivity of the nanocomposites. The [111] planes, characterized by their rich concentration of active sites, significantly drive this mechanism. Consequently, the unique interplay between Co_3_O_4_ nanoparticles, [111] plane exposure, and electron beam radiation establish an intricate yet effective pathway for boosting conductivity within the XLPE/Co_3_O_4_ nanocomposites.

## Conclusion

This study demonstrates the multifaceted impacts of electron beam irradiation on Co_3_O_4_-based nanocomposites and highlights the potential for tailoring their properties through controlled manipulation. The findings underscore the promise of such nanocomposites in applications ranging from enhanced electrical conductivity to potential usage as solid electrolytes, while shedding light on their structural, optical, magnetic, and electrical transformations under electron irradiation.

In the pursuit of developing nanocomposites, nanosized ferromagnetic Co_3_O_4_ particles were synthesized using the sol–gel method and subsequently incorporated into the XLPE matrix. As explored by ESR, the electron beam irradiation of Co_3_O_4_ nanoparticles led to increased free un-paired electrons. This phenomenon suggests that the irradiation process has induced oxidation of surface Co^2+^ ions which leads to partial elimination of oxygen vacancies, freeing the trapped electrons and generating an elevated number of unpaired electrons, confirmed by XPS and PL techniques, respectively. As the oxygen vacancies are mainly responsible for the weak ferromagnetic behavior of Co_3_O_4_ nanoparticles, the irradiated Co_3_O_4_ nanoparticles at a dose of 30 kGy showed lesser saturation magnetization (0.508 emu/g) than the un-irradiated Co_3_O_4_ nanoparticles (0.538 emu/g).

In investigating the magnetic behavior of XLPE/Co_3_O_4_ nanocomposite, prepared via melt extrusion, a weak ferromagnetic behavior is observed which is characterized by low coercivety ~ 175.72 G which further decreased to 135.18 G upon irradiating the nanocomposite at a dose of 30 kGy of electron beam. However, both values are higher than that of the un-irradiated Co_3_O_4_ nanoparticles ~ 112.57 G. The highly increased dislocation lines density initiated during the polymerization process could cause pinning of the domain wall. So, more external field would be exerted to unpin the domain wall from its pinned position and accordingly, the polymeric nanocomposite before and after irradiation showed greater coercivities. The room temperature ferromagnetic behavior with relatively low coercivity values of our prepared nanocomposite before and after irradiation suggests using it as soft magnets which find applications in spintronics. On the other hand, comprehensive optical and dielectric analyses reveal the semiconducting nature of the nanocomposite, evidenced by the range of its two indirect optical band gap (2.06 and 3.55 eV), and high dielectric permittivity. The influence of varying electron beam doses on the electrical properties of the nanocomposite has also been explored. A marginal yet notable augmentation in AC and DC conductivity is observed post-irradiation. Incorporating Co_3_O_4_ nanofillers into XLPE has boosted its conductivity from 0.133 × 10^–7^ S/m to 2.198 × 10^–3^ S/cm which is ascribed to the ionic conductivity of the resultant polymer nanocomposite. Upon exposure to a 30 kGy dose of electron radiation, this conductivity further increases to 3.614 × 10^–3^ S/cm, showcasing the potential utility of this nanocomposite as a solid electrolyte.

## Data Availability

The data that support the findings of this study are available from the corresponding author on request.
